# Conducting polymers as electron glasses: surface charge domains and slow relaxation

**DOI:** 10.1038/srep21647

**Published:** 2016-02-25

**Authors:** Miguel Ortuño, Elisa Escasain, Elena Lopez-Elvira, Andres M. Somoza, Jaime Colchero, Elisa Palacios-Lidon

**Affiliations:** 1 Dep. de Física - CIOyN, Universidad de Murcia, E-30100 Murcia, Spain; 2 Dep. Surfaces and Coatings, Instituto de Ciencia de Materiales de Madrid - CSIC (Campus Cantoblanco), E-28049 Madrid, Spain

## Abstract

The surface potential of conducting polymers has been studied with scanning Kelvin probe microscopy. The results show that this technique can become an excellent tool to really ‘see’ interesting surface charge interaction effects at the nanoscale. The electron glass model, which assumes that charges are localized by the disorder and that interactions between them are relevant, is employed to understand the complex behavior of conducting polymers. At equilibrium, we find surface potential domains with a typical lateral size of 50 nm, basically uncorrelated with the topography and strongly fluctuating in time. These fluctuations are about three times larger than thermal energy. The charge dynamics is characterized by an exponentially broad time distribution. When the conducting polymers are excited with light the surface potential relaxes logarithmically with time, as usually observed in electron glasses. In addition, the relaxation for different illumination times can be scaled within the full aging model.

Conducting and semiconducting polymers have been proposed as the fourth generation of polymeric materials^1^. Due to their easy processability, high tunability and low-cost production, they have a great potential for a variety of applications. Plastic electronics devices, such as organic field-effect transistors, organic light-emitting diodes, and organic solar cells, are already fabricated and commercialized^2^. Apart from their technological applications, conducting polymers offer a wealth of interesting and challenging basic phenomena from the fundamental point of view. Typical conducting polymers are semi-crystalline in the sense that locally the chains align in an ordered structure forming nano-crystallites which are surrounded by amorphous regions. This nanostructure determines the intra and inter-chain interactions that govern the electronic properties of the material^3^,^4^,^5^.

It is well accepted that, due to the high degree of disorder, in polimeric materials the conduction occurs by phonon assisted hopping between localized states, involving the formation of polarons or bipolarons^6^,^7^. Although it has been profoundly studied for the last decades, the complexity of conducting polymers prevent a full consensus about the underlying conduction mechanisms and in particular the way they relax after excitation far from equilibrium. Interactions between carriers are likely to be significant enough to affect their conduction mechanism, as evidenced by the *T*^−1/2^ dependence of the logarithm of the conductivity in the variable-range hopping regime^8^, and makes the conduccting polymers excellent candidates to be electron glasses.

Electron glasses are systems with states localized by the disorder and with long-range Coulomb interactions between carriers. Disorder produces localization of the wavefunctions, which in turn results in a lack of screening and an increase in the importance of Coulomb interactions. Very slow relaxation rates are commonly observed in these systems due to the exponential dependence of the transition rates on hopping length and energy and to the many-valley structure of the phase space produced by the interactions^9^.

Typical glassy phenomena observed in electron glasses include a slow logarithmic decrease of the conductivity^10^,^11^, a memory dip^11^,^12^,^13^ and aging^12^,^14^,^15^. These phenomena have been observed in a great variety of materials, such as indium oxides^10^, granular metals^11^, thin metal films^13^,^16^, and recently GeSbTe films, where glassy phenomena coexist with persistent photoconductivity^17^.

Electron glasses have been excited with electromagnetic radiation, and for high enough frequency, slow relaxation is observed^10^,^17^,^18^. These results are consistent with the following picture. At equilibrium, charges are arranged in low energy configurations that minimize the Coulomb energy and present complex correlations between carriers and a Coulomb gap in the single-particle density of states^19^,^20^. If the radiation frequency exceeds the Coulomb gap energy, charges are randomized and reestablishing the equilibrium configuration is usually a very slow process.

Slow logarithmic relaxation of the electrochemical doping potential was observed over nine orders of magnitude in polymeric materials^21^. A dependence of the logarithmic shift with scan rate and aging phenomena were also observed. The universal features of slow relaxation were attributed to a hierarchical series of processes, following ideas from kinetic studies of the decay of persistent photoconductivity in semiconductor structures^22^,^23^.

Slow relaxation in the photoconductivity in poly(phenylenevinylene) (PPV) films was studied by Lee *et al.*^24^. A roughly logarithmic behavior was observed, as well as an *ω*^0.66^ dependence of the photoconductivity with chopping frequency, which was explained again in terms of hierarchical processes working in series. Logarithmic relaxation over many orders of magnitude was observed in photoinduced conductivity in organic field effect transistor^25^. Under illumination, the photoinduced excess current also increases logarithmically.

All experiments mentioned describe conductivity at the macroscopic scale. Some mesoscopic properties have also been analyzed^13^,^26^,^27^, but the nanoscale has never been reached in electron glass studies. The scanning force microscopy (SFM) technique offers the double opportunity of being able to observe nanoscopic properties of interacting systems and to follow the relaxation behavior for a quantity other than conductivity. In this letter, we use the scanning Kelvin probe microscopy (SKPM) to measure the surface potential (SP) in order to explore a possible, very natural explanation for slow relaxation in conducting polymers based on electron glass ideas. Firstly, we study the SP distribution in equilibrium and see a well defined domain structure with spatial and temporal correlations fully compatible with the electron glass model. Secondly, the sample is excited by irradiation with green light for a certain time and we monitor how the sample SP relaxes to equilibrium once the excitation is switched off. We have observed a logarithmic behavior characteristic of glassy interacting systems.

## Results

### Nanoscale charge domains

The nanoscale SP of MEH-PPV thin films has been studied by means of SFM movies (see Materials and Methods). These movies are a powerful tool to investigate dynamic properties at high resolution and have been previously used to study, for example, single atom diffusion^28^ or phase transitions^29^. Here we utilized them to study the dynamics of electrons on the surface of thin polymer films.

#### Domain characterization

**Figure 1:**
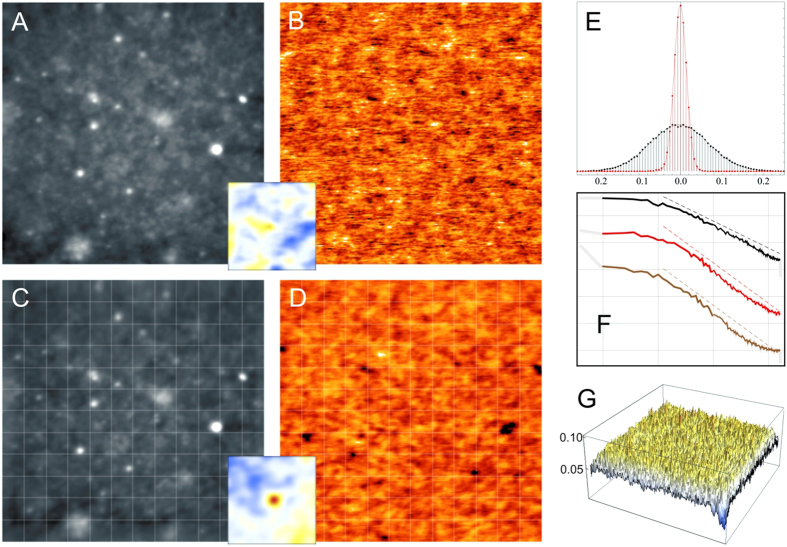
(**A**) Topography image (*z* scale 6 nm, lateral scale: 1500 nm), and (**B**) the corresponding SP image (*z* scale ± 150 mV) of a typical MEH-PPV thin film, together with their cross-correlation (inset between them). (**C**) Average topography image (*z* scale 6 nm, lateral scale: 1500 nm), and (**D**) average SP image (*z* scale ± 50 mV) of a film of 91 frames at a rate of 20 minutes/frame. Their cross-correlation is shown in the inset between them. (**E**) Histogram of the SP in (**B**) (black) and in (**D**) (red). (**F**) Power spectral density of the SP of a single frame (black), of the average image (red) and of the topography (brown); the straight lines are a guide to the eye and have slopes 2 (black), 3 (red) and 3 (brown). (**G**) standard deviation image of the SP, *δ*_*S*_(*x*, *y*).

Figure 1A,B show a typical topographic and SP frame, respectively, extracted from a movie (20 min/frame)(Video S1 and S2 Supplentary Information). The features observed in the topographic and the SP images are essentially independent, as can be seen directly or deduced from the lack of a central peak in their cross-correlation image (inset between the two figures). The roughness of the topographic image is about 1 nm and the typical lateral size of the topographic features is about 40 nm. The histogram of the SP (black curve in Fig. 1E) shows a Gaussian distribution, with a standard deviation of *σ*_*S*_ = 71 mV. The mean SP domain size is about 50 nm. It has been checked that all frames have the same statistical properties. In particular, *σ*_*S*_ is about 70 ± 2 mV in all frames, and surprisingly about three times larger than the thermal energy *kT* = 25 meV at room temperature.

To further explore the properties of the films, the average of all (forward) topography and SP (*S*_av_(*x*, *y*)) frames of a movie (Fig. 1C,D respectively)has been calculated using Eq. (3). The morphology of the sample barely changes during the whole movie. On the contrary, the SP domains change appreciably from frame to frame. This change is not due to instrumental noise (≈10 mV), as proved by the fact that the forward and backward scan directions basically coincide (Fig. S1 Suplementary Information). In addition, the average SP image presents a stable and well-defined structure, implying that it is not due to random fluctuations, but to real electronic properties of the sample. The histogram of the average SP, *S*_av_(*x*, *y*), is shown in Fig. 1E (red curve). The standard deviation of *S*_av_(*x*, *y*) is 14 mV, and thus much smaller than the standard deviation of single frames (70 mV). Therefore, the average charge of a domain must be much smaller than its typical instantaneous charge. The cross correlation between the average topography and the average SP (inset between Fig. 1C,D) shows a small peak in the center (of height 1/20) indicating a weak correlation between the average SP and the topography, which can hardly be resolved for individual frames.

The standard deviation over time *δ*_*S*_(*x*, *y*) (Eq. 4) is shown in Fig. 1G. *δ*_*S*_(*x*, *y*) is essentially constant (≈70 mV) and shows no significant spatial structures. This constant is quite large, showing that the SP has a high variability over time independent of *x* and *y*. Interestingly, this value is similar to *σ*_*S*_ in a typical single frame.

#### Time correlations

**Figure 2:**
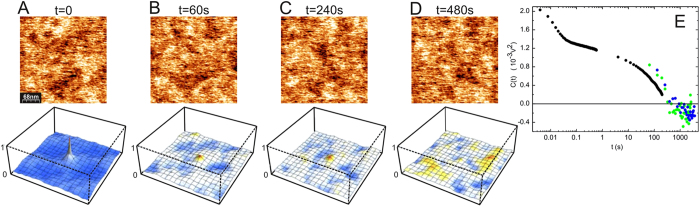
Top panel. (**A**–**D**) four SP frames from a movie (60 seconds/frame) taken at the times indicated. Bottom panel. 3D plot of the normalized correlation of corresponding SP frame with the first frame. (**E**) Time correlation function *C*(*t*) on a semilogarithmic scale. Black symbols correspond to measurements performed on a fixed sample position, while green and blue symbols correspond to movie frames acquired at 1 and 2 minutes/frame (Video S4 and S6 respectively).

In order to study dynamic properties we compare successive movie frames through their cross-correlation. Frames in Fig. 1 were taken at a rate of 20 minutes/frame to minimize noise and the cross-correlation between consecutive frames is basically zero. To increase time resolution, fast images were acquired (from 15 to 120 seconds/frame). Figure 2A–D shows four representative frames extracted from a fast movie (60 s/frame) corresponding to different times (Video S4 Suplentary information). The autocorrelation of the first frame (*t* = 0) together with cross-correlation between the first frame and successive frames (Fig. 2A–D bottom panel) show that the cross-correlation decays over a few frames, corresponding to a characteristic decay time of about three minutes.

To analyze further the dynamical response of the system, we measure the SP evolution with time at a fixed point. In this way we are able to explore time scales from tens of milliseconds (limited by the response of the microscope) to several minutes (limited by the drift of the apparatus, which cannot be corrected for a fixed point). From these data, we calculate the time correlation function *C*(*t*), (Eq. 4), plotted in Fig. 2E. One can appreciate that the scale of characteristic times is very wide, spanning more than four decades. This wide spread of characteristic times is usually found in glasses and is ascribed to the existence of a hierarchical set of processes. To confirm that the overall behavior at different timescales is consistent, *C*(*t*) obtained for two movies acquired at different speeds (Video S4 and S6) has been included (Fig. 2E green and blue symbols). The agreement is good taking into account that the data were acquired at different speeds and on different samples. The fast decay at short time scales is related to the dynamical response of the apparatus.

#### Spatial correlations

**Figure 3:**
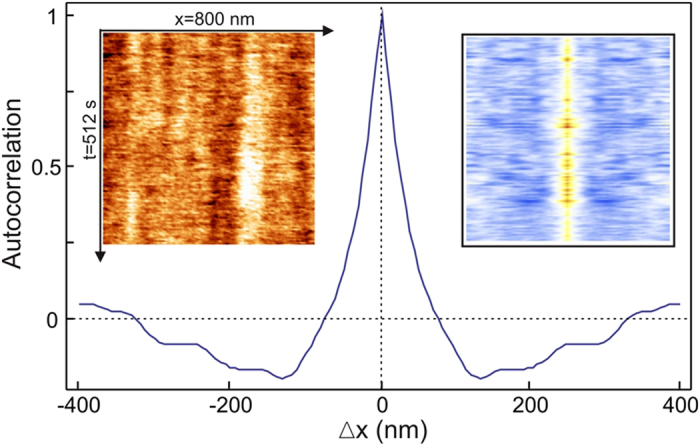
The left inset shows the SP for successive scans along the same line, the horizontal axis is position along the line and the vertical axis is time. The right inset is the autocorrelation function of the left inset. The main panel is the average over time of the autocorrelation function of the SP along the line.

The study of spatial correlations is a complicated problem since dynamic time scales are comparable or even faster than data acquisition times. Very fast scans are crucial in order to minimize the effects due to temporal evolutions. To overcome this difficulty as much as possible, the SP was measured along the same line for a series of scans. An example is shown in the left inset of Fig. 3, where the horizontal axis is the position along the line and the vertical axis time. One can appreciate vertical bright and dark lines, corresponding to charge domains that are stable on the timescale of seconds to minutes. It can be clearly seen that most domains change their state and some of them change rather suddenly at a given instant probably due to charge jumps. The autocorrelation function for each horizontal line of the SP data is shown in the right inset of Fig. 3. The line average of this autocorrelation function (main panel of Fig. 3) shows a central peak with a negative region. The width of the central peak, around 40 nm, corresponds to the lateral size of the domains.

#### Physical interpretation in terms of an electron glass

The first point that should be addressed is the size of the SP spatial fluctuations (*σ*_*S*_ = 71 mV), roughly a factor 3 larger than the thermal energy. This can be naturally explained within the electron glass model, since in this model site energy fluctuations are much larger than *kT*^30^,^31^. Transition energies are of the order of *kT*, and are equal to the difference in site energy minus the interaction energy, which can be quite large. Site energy fluctuations can be appreciably larger than transition energies, thus larger than *kT*. The variation of the SP may be due to disorder energy of the intrinsically disordered polymeric material, to possible defects/trap sites as well as to a different electrochemical potential of crystalline/amorphous regions of the semiconducting polymeric material and, finally, to the Coulomb energy *E*_coul_ of a charge confined to a region of radius *R*. Assuming that domains are singly charged, for a radius *R* = 25 nm the Coulomb energy is 60 meV, quite compatible with the observed variations of the SP.

The spatial fluctuations of the time-averaged SP image, around 14 meV, are much smaller than single frame fluctuations, which indicates that the fluctuations of the average SP are mainly due to changes in the local contact/chemical potential. Each frame corresponds to a particular arrangement of charges. These charges evolve in a disordered mean effective energy landscape generated by local variation of the material properties and/or some mean effective electron configuration.

What is quite remarkable - in our opinion - is that some electronic events occur on very large timescales of up to minutes, and are thus observable by SFM techniques. The electron glass model is an excellent candidate to explain this, since frustration and interactions can produce characteristic times exponentially distributed. The measurement time of SFM is of the order of a millisecond, therefore any dynamics faster than this time is averaged. In this sense, the observed domain radius should be considered as an upper bound of the real localization length of the charges. Nevertheless, this does not invalidate the previous argument relating domain size and SP fluctuations, since both quantities will be averaged in a similar way.

In order to get further insight into the charge domain dynamics, we have performed the same experiment in a MEH-PPV sample that was previously irradiated with blue light for a short time period (see Supporting Information for details). It is well known that blue light photo-induces MEH-PPV degradation, decreasing the *π*-bond conjugation length and therefore the material conductance^32^,^33^. Comparing this degraded sample with the non-degraded one, it is found that although in the degraded sample a charge domain dynamics is still present, the corresponding changes occur less frequently. The decay time of the cross-correlation *C*(*t*) increases from about 3 min to about 15 min (see Fig. 2 of Supporting Information). Those results fully support the idea that we are observing hopping dynamics, and that a decrease in conductance is correlated with slower domain dynamics.

The existence of a region where the autocorrelation function is negative is another indication of the importance of interactions, since a region with a given charge is more likely to be near a region with opposite charge. This supports the applicability of the electron glass model to MEH-PPV, which is strongly reinforced by the relaxation experiments that we report below.

Finally, we recall that the SFM technique probes mainly surface properties, therefore the images discussed reflect electron dynamics within a relatively thin surface layer. From our experiments we cannot conclude if the phenomena that we are observing is a pure 2D surface effect or a surface projection of bulk effects. In any case, the dimensionality of the electron glass does not affect the interpretation and relevance of our results.

### Slow relaxation

A suitable tool to study complex systems is to drive them out of equilibrium and to study how they return to equilibrium. To achieve a proper sample excitation the “two pass” method^34^ is used. In this protocol the sample is illuminated with several “on”/“off” cycles until the light is completely switched off and the system is allowed to relax. By recording the SP, *S*(*t*), along the whole experiment, relevant information from the excited state as well as from the relaxation processes can be inferred.

**Figure 4:**
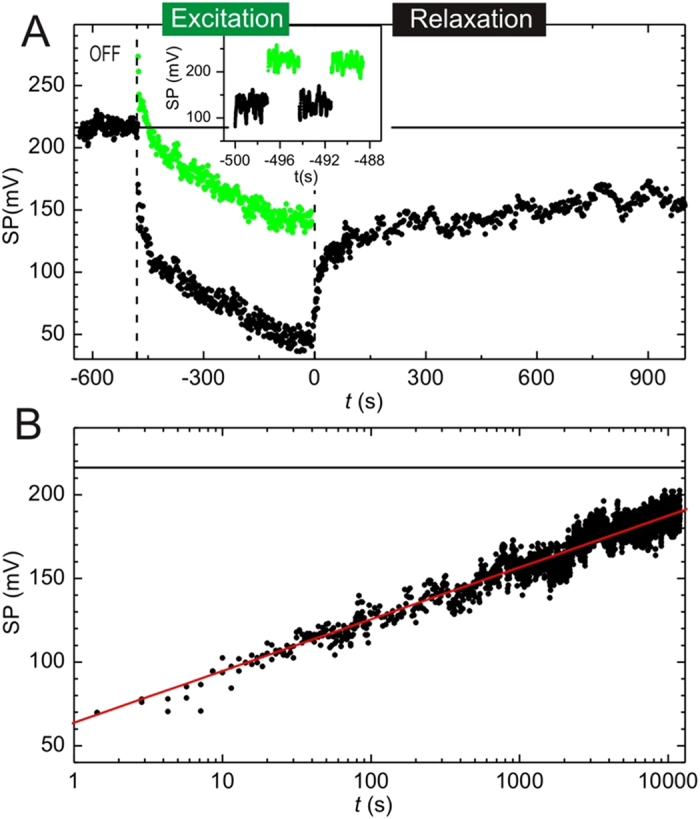
Top panel: average SP as a function of time when light is switched on and off periodically in the interval marked by the two vertical lines. Green dots correspond to measurements during the “on” period, while black dots to those with light off. Bottom panel: the same data as before after the light has been definitely switched off versus the relaxation time on a logarithmic scale. The straight line is a logarithmic fit to the data.

Figure 4A shows the time evolution of *S*_av_(*t*) in a typical MEH-PPV thin film excitation experiment. The horizontal black line shows the average SP of a sample region that has not been previously illuminated. This average SP value characterizes the initial state and will be used as reference to evaluate photo-induced excitations. As shown in the inset of Fig. 4A, which is an enlargement of the main figure, during the “two pass” excitation protocol, the light is switched on and off alternatively in periods of 1.5 s. Green and black dots represent SP values obtained under illumination and in darkness, respectively. It should be noticed that processes faster than the scanning time of a line cannot be resolved. As shown in ref. 34, it is observed that the average *S*(*t*) increases when the light is “on”, as expected for a p-type material^35^. However, *S*(*t*), instead of returning to its equilibrium value during the “off” part of the illumination protocol, decreases to smaller values. As more light excitation cycles are applied, the SP for both with and without illumination periods decreases slowly. This indicates that relaxation in the “off” part of each cycle is not complete and the system experiences a higher and higher degree of excitation as more cycles are applied.

Once the light is definitively switched off, the SP slowly tends (relaxes) to its initial value. This relaxation behavior is qualitatively similar (with opposite sign) to the excitation curve, a feature usually found in electronic glasses^10^,^14^,^36^. To further analyze the relaxation tendency, data has been plotted in Fig. 4B on a logarithmic scale with the origin of time taken at the instant at which light is completly switched off. Relaxation of the SP to its equilibrium value is roughly logarithmic over four decades of time. Hence, we are clearly dealing with a very slow relaxation process. The rate of relaxation decreases when the intensity of the light increases. A similar effect occurs in GeSbTe in the presence of persistent photoconductivity^17^, and is associated to an increase in carrier density.

The logarithmic relaxation of the average SP implies the existence of an exponentially broad distribution of relaxation times^37^,^38^. This type of distributions is obtained whenever the transition rates are exponential functions of smoothly distributed random variables, as naturally occurs in hopping systems^39^. In these systems, states are localized and the transition rates for one-electron hops are given by^9^


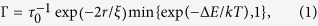


where *τ*_0_ is a typical phonon time, *r* the hopping distance, *ξ* the localization length, and Δ*E* the transition energy. For interacting systems, simultaneous many-electron hops are possible and this equation is still a valid approximation considering *r* as the sum of all the hopping distances. Previously, slow relaxation in organic polymers was explained in terms of hierarchical processes with an increasing spatial separation between the negative charges at the surface and the positive charges in the bulk^25^. This model cannot explain the rich variety of phenomena that we see in our systems. We think that the existence of the domain structure of the SP together with the logarithmic relaxation favor an explanation in terms of electron glasses. Slow logarithmic relaxation phenomena have been observed in a great variety of systems, grouped under the name electron glasses, as they all conduct by hopping and interactions between carriers are believed to be important. The details of the mechanism responsible for slow relaxation in electron glasses are not known. Nevertheless, there is a growing consensus that energy relaxation requires a hierarchical series of processes involving an increasing number of particles participating in a hop^40^, whose characteristic times grow exponentially with this number. The existence of SP domains ensure the presence of charges and the fact that they fluctuate in time proves the relevance of hopping processes. Additionally we argue that average SP should be correlated with the macroscopic conductance, reinforcing our idea that we are studying the same phenomena that has been observed in electron glasses. After illumination, in a highly excited state, there are many holes below the chemical potential as well as many electrons above it. This implies that there is a shift in the chemical potential with respect to equilibrium (as long as the single-particle density of states is asymmetric) and, at the same time, there is a increase in conductance because there are many more possible excitations. As the system relaxes, changes in the conductance should be correlated to changes in the average SP. Our observations and the presence of aging (to be discussed in the next section) are difficult to explain with previous models of slow relaxation in organic polymers, while they are naturally explained with the electron glass model.

### Aging

**Figure 5:**
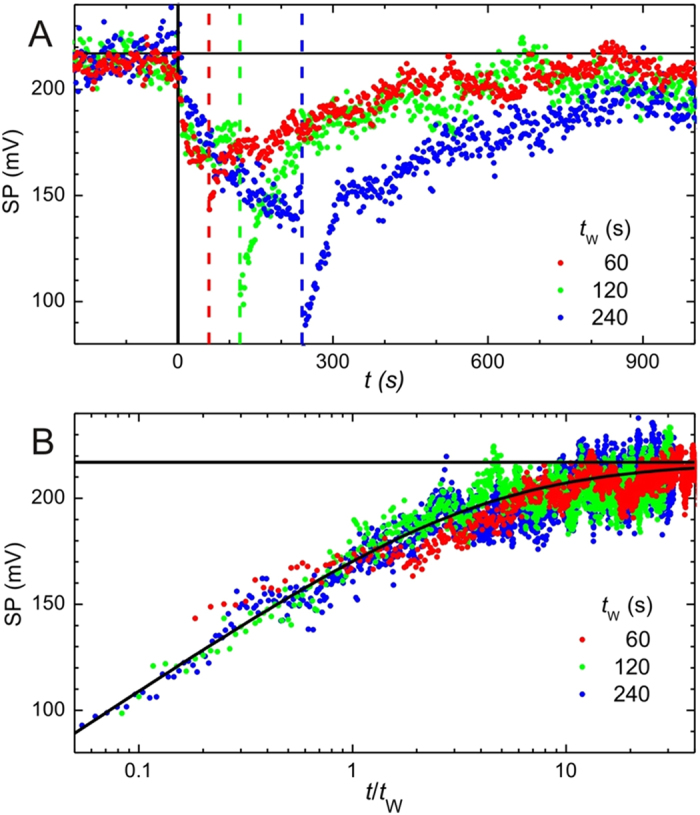
Top panel: average SP for three different illumination times *t*_*w*_ = 60 s (red), 120 s (green) and 240 s (blue) versus time measured from the instant in which light is switched on (vertical solid line). The dashed vertical lines show the time at when the light is switched off. The horizontal line is the average SP at equilibrium. Bottom panel: same data as in top panel as a function of *t*/*t*_*w*_ on a logarithmic scale. The continuous curve is a theoretical prediction, Eq. (2).

Besides slow relaxation, other glassy properties observed in many circumstances and systems are memory effects and aging, which we now analyze for our systems. As explained in the SI sample excitation, in our case the aging protocol consists in illuminating the sample during a certain period of time *t*_w_ (called waiting time in the literature) and then switching off the light, letting the sample relax. In this protocol a light intensity smallerthan for the study of the relaxation behavior is used. In Fig. 5A the evolution of the SP is presented for three different values of waiting time *t*_w_. The vertical solid line indicates the moment at which the light is switched on, while the dashed lines correspond to the moment when the light is switched off for each *t*_w_. It can be seen that during the excitation, the three SP curves approximately overlap. The horizontal solid line shows 〈*S*〉_eq_, the initial SP value of the sample before any illumination which is used as reference value for the relaxed state.

To study the dependence of the SP relaxation with *t*_w_, the data are represented on a logarithmic scale as a function of *t*/*t*_w_ taken the time origin (*t* = 0) as the moment when the light is switched off (Fig. 5B). It is clear that to a good approximation, the three curves collapse. In fact, the overlap curve can be fitted to the equation.


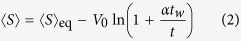


where 〈*S*〉_eq_ is the average SP before illumination, and *V*_0_ is some characteristic excitation surface potential, equals 67 mV in our case. This expression was obtained in the context of relaxation in electron glasses^39^,^41^, with the factor *α* = 1. Factors *α* different from one, ranging between 1 and 10, have been previously observed in electron glasses. For example, in the so-called F protocol, where the sample is driven out of equilibrium by applying a strong non-ohmic electric field^42^. The understanding in the electron glass community is that when the “waiting” period corresponds to relaxation to a new equilibrium state, typically created by changing the gate voltage, one has *α* = 1, while when the “waiting” period corresponds to a truly excitation phase, as is the case here and in the F protocol, values of *α* larger than one are found.

In Fig. 5A we can also observe that the behavior of the SP when the light is on, the excitation phase, is roughly symmetric to the relaxation behavior, as with the previous relaxation protocol, and a trend often seen in electron glasses^10^,^14^,^36^.

It should be highlighted that the present aging protocol leads to important memory effects that prevent a high quality logarithmic relaxation behavior, and therefore it cannot be used for larger *t*_w_. In Fig. 5A, one can appreciate in the sample that has been excited for a longer period of time (blue symbols) a small plateau in the SP. This plateau is associated with an incipient memory effect of the excited state. To achieve high quality logarithmic relaxation curves, the “two pass” protocol shown in the previous subsection and higher light intensities should be used. If higher light intensities are used with the present protocol, scaling with *t*_w_ is still possible, but full aging is lost and one observes subaging, i.e., a scaling in terms of the variable 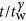, with the exponent *γ* smaller than one.

## Conclusions

Our SFM studies on the MEH-PPV conducting polymer film show strong evidence of the formation of an electron glass on the surface of the material. This evidence includes the presence of domains on the SP, uncorrelated with topography, and showing self-repulsion, indicative of the relevance of interactions. The fluctuations of SP are compatible with variations of the Coulomb energy of a single charge over the distance between domains. At the same time, the fact that the SP fluctuations are larger than *kT* and that time correlations are dominated by a broad distribution of characteristic times can be naturally explained within the electron glass model^30^,^39^.

We have studied the domain structure and dynamics in low degraded (less conducting than non-degraded MEH-PPV). The overall tendency of domain sizes and SP fluctuations is the expected one from our model. The lower the conductance, the smaller the domain size and the larger the variation of the SP. Quantitative comparison between domain size and SP fluctuations in a large enough range of conductance, is outside the scope of the present work and will be addressed in a future work.

The domain dynamics in degraded and non-degraded samples shows similar a similar overal behaviour but a slower dynamics. In addition, it has been previously reported that highly-insulator polymers also present surface charge domains but with no dynamics^43^. These results are consistent with the interpretation that we are observing hopping phenomena.

In addition, after excitation with light, the samples show very slow relaxation of the average SP. Under appropriate excitation conditions, logarithmic relaxation, characteristic of electron glasses, is observed over four decades of time. Relaxation rate decreases with light intensity and so with carrier density. Full aging is also observed: relaxation curves for different excitation times *t*_w_ can be overlapped when plotted versus *t*/*t*_w_, and the overall relaxation curve follows the prediction for electron glasses, Eq. (2).

Finally, we expect the formation of surface charge domains in hopping-conduction materials to be a very general phenomena. Up to now electron glasses have been studied experimentally through macroscopic properties of the material, in particular conductivity relaxation. We have shown here that the SKPM technique is a powerful tool to monitor and study fundamental properties of disordered systems at the nanoscale and, possibly, at the single charge level. The relation between macroscopic conductivity and the microscopic behavior of slow relaxation is a difficult, but very important issue. In the future we will combine classical macroscopic techniques, mainly based on the analysis of conductivity, with nanoscale techniques to address this problem and to discern between different models for slow relaxation.

## Materials and Methods

### Sample preparation

Poly[2-methoxy-5-(2-ethylhexyloxy)-1,4-phenylenevinylene] (Mn 150,000–250,000 Sigma-Aldrich) (MEH-PPV) thin film samples were prepared by spin-coating (2500 rpm) from chloroform solution (30 mg/ml) on indium tin oxide coated PET (ITO:PET) (Sigma Aldrich) previously cleaned with isopropanol. Thin MEH-PPV films with an average thickness of 200 nm are obtained. The ideal chain model gives a gyration ratio of 6–8 nm. Further description of the sample surface can be found in ref. 34.

### SFM Measurements

SFM experiments were performed at room temperature and ambient conditions. For non-optical experiments a Nanotec Electronica SFM setup with a control unit equipped with a PLL/dynamic measurement board was employed. Signal to noise ratio is a limiting factor in our experiments, since high spatial resolution, high energy resolution and large bandwidth are required. In order to have thermal noise-limited performance^44^,^45^, a monomode polarization preserving fiber is used as light source for the laser detection system. Topography images were acquired working in non-contact dynamic mode using the frequency shift as feedback parameter (FM-DSFM). Frequency modulation SKPM (FM-SKPM) with an AC modulation bias of *U*_ac_ = 500 mV at 7 kHz was used to measure the local surface potential (SP). Further details of the SKPM set up and SFM working modes are described elsewhere^45^. Platinum coated silicon tips (Budget Sensors), with nominal force constant of 3 N/m and resonance frequency of 75 kHz, were used.

### Data processing

For the study of equilibrium properties, successive SFM images –movies– have been acquired with constant acquisition parameters. Precise alignment of all movie frames is fundamental, since the processing algorithms need to compare a particular (*x*, *y*) position in one frame, with the same (physical) position in other frames. Thermal drift and the length of the movies (up to days) imply that the mechanical setup varies over time and different frames are misaligned. To correct this misalignment and process the images the free WSxM software is used^46^. This software computes the cross-correlation between images and finds the position of the maximum of the cross-correlation corresponding to the offset between images of the movie. This offset defines a drift path which allows computing a drift-free movie.

From these drift-free movies, all relevant information is calculated: single cross-correlation between frames taken at different times, cross-correlation movies obtained by calculating the cross-correlation of each individual frame with a selected frame of the movie (usually the first) and finally average frames. These average frames are calculated by taking the average at each point of any quantity through the whole movie. In particular, the average of the SP is:


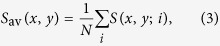


where the index *i* runs over all the frames *S* (*x*, *y*, *i*) of the movie. The standard deviation of the SP at each point is


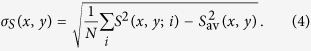


The well-defined averages of topography and error signal (See Fig. S2 Suplementary information) prove that the drift correction algorithm aligns the frames of a movie to within a few image points; an accuracy of 2–3 pixels is estimated, which is of the order than the spatial resolution of our experiments. We note that exactly the same alignment protocol is applied to all SFM channels.

To analyze dynamical properties, we introduce the time correlation function *C*(*t*), defined as


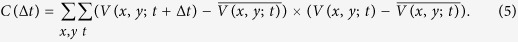


### Sample excitation

Experiments involving sample excitation with light have been carried out in a home- build SFM implemented in an inverted optical microscope (Nikon Eclipse). As explain elsewhere, this set up allows to illuminate the sample in a controlled way and to measure the topography and SP in darkness as well as under illumination^47^. In the present work, the polymer is illuminated through the transparent ITO:PET electrode with green light (*λ* = 535 nm) at an intensity between 2 and 7 × 10^16^ photons/(s cm^2^). This wavelength lies within the absorption band of the MEH-PPV inducing exciton generation without degrading the polymer. Under these conditions, only photo-physical reversible processes take place^34^,^48^. The illuminated area (≈300 *μ*m^2^) is about two orders of magnitude larger than the SFM image size (1–16 *μ*m^2^).

Sample light-excitation experiments have been performed in three steps. First several SKPM images are acquired in darkness for a long period of time (from several hours to days) on a sample region that has been never illuminated before. Secondly, the polymer is illuminated for a certain period of time. Finally the light is completely switched off and the system is left to relax to the equilibrium. During the whole process, the local SP evolution is monitored to study the time evolution.

Two different protocols have been used for sample illumination. In relaxation experiments, the line by line “two pass method” has been used. The detailed working principle and data processing are explained elsewhere^34^. Briefly, in this method the light is pulsed in short “on/off” cycles by scanning each line twice. The first trace is performed in darkness, while the second pass under is illumination. Then the tip moves to the next horizontal line and the process is repeated. In this way, two SKPM images are recorded simultaneously, one in darkness (“off” SKPM image) and the other one under illumination (“on” SKPM image). For the aging experiments a continuous illumination protocol has been used. That is, the light is switched on and the sample is illuminated for a certain period of time (*t*_w_).

To obtain the SP evolution the SKPM images are processed as follows: the mean SP of each horizontal image line is computed and plotted as a function of time. Sample excitation experiments can be performed at a fixed sample position (*x*_0_, *y*_0_), at a fixed sample line (*x*, *y*_0_) or in a sample region (*x*, *y*). In this work we have used the latter two cases. It should be noticed that in these cases the SKPM images include not only time dependence information but also spatial information. Therefore the spatially averaged nanoscale behaviour of the SP as a function of time is obtained.

## Additional Information

**How to cite this article**: Ortuño, M. *et al.* Conducting polymers as electron glasses: surfacecharge domains and slow relaxation. *Sci. Rep.* **6**, 21647; doi: 10.1038/srep21647 (2016).

## Supplementary Material

Supplementary Information

Supplementary Video S1

Supplementary Video S2

Supplementary Video S3

Supplementary Video S4

Supplementary Video S5

Supplementary Video S6
