# Supplementary material for: Conducting polymers as electron glasses: surface charge domains and slow relaxation
Source: Sci Rep. 2016 Feb 25;6:21647. doi: 10.1038/srep21647 (PMC4766496; doi:10.1038/srep21647)
Supplement: Supplementary Information [file srep21647-s1.pdf]

Conducting polymers as electron glasses: surface charge domains and slow relaxation

Miguel Ortuño[1], Elisa Escasain[1], Elena Lopez-Elvira[2], Andres M. Somoza[1], Jaime Colchero[1], and Elisa Palacios-Lidon[1,*]

[1]Dep. de Física - CIOyN, Universidad de Murcia, E-30100 Murcia , Spain
[2]Dep. Surfaces and Coatings, Instituto de Ciencia de Materiales de Madrid - CSIC (Campus Cantoblanco), E-28049 Madrid, Spain
[*]elisapl@um.es

ABSTRACT

The surface potential of conducting polymers has been studied with scanning Kelvin Probe microscopy. The results show that this technique can become an excellent tool to really 'see' interesting surface charge interaction effects at the nanoscale. The electron glass model, which assumes that charges are localized by the disorder and that interactions between them are relevant, is employed to understand the complex behavior of conducting polymers. At equilibrium, we find surface potential domains with a typical lateral size of 50 nm, basically uncorrelated with the topography and strongly fluctuating in time. These fluctuations are about three times larger than thermal energy. The charge dynamic is characterized by an exponentially broad time distribution. When the conducting polymers are excited with light the surface potential relaxes logarithmically with time, as usually observed in electron glasses. In addition, the relaxation for different illumination times can be scaled within the full aging model.

Suplementary Information

Domains characterization

The SP domains change appreciably from frame to frame. This change is not due to instrumental noise ($\approx$ 10 mV), as proved by the fact that the forward and backward scan directions basically coincide (Fig.1).

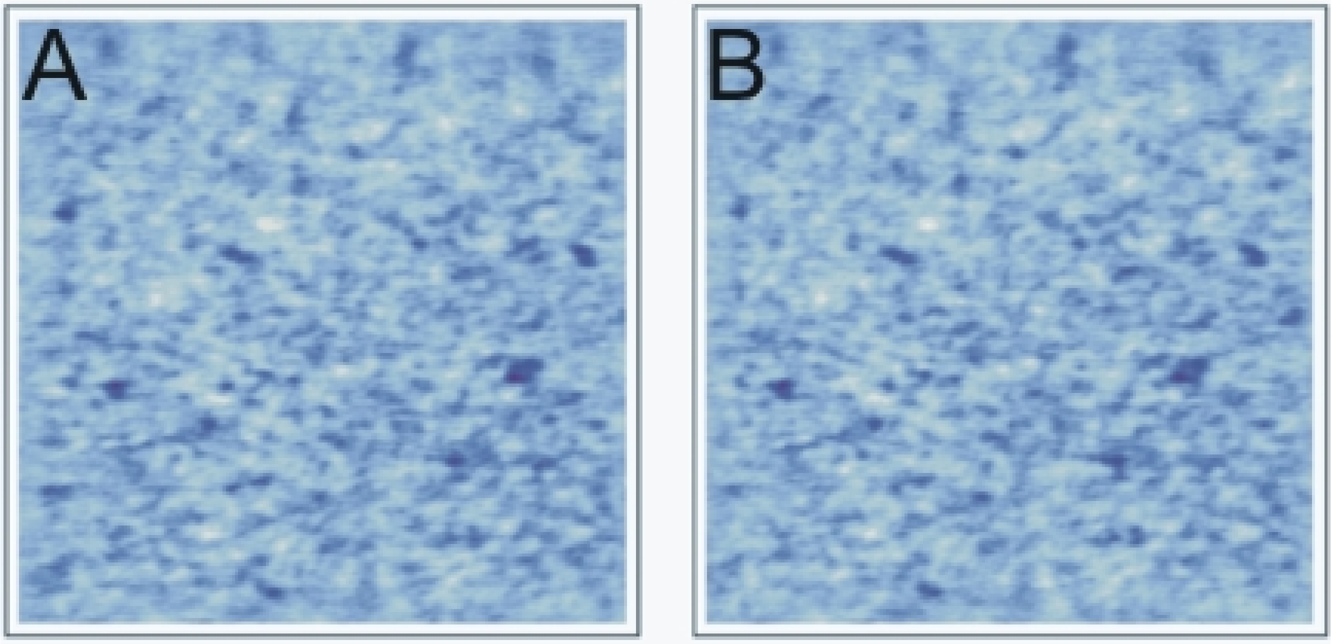

Figure 1. (A) Average forward SP image and (B) Average backward SP image obtained from a movie of 91 frames (20 min/frame).

Degraded MEH-PPV sample

It is well known that blue light photo-induces MEH-PPV degradation, decreasing the π-bond conjugation lenght and therefore the material conductance. To study the effects of thegradation on the charge domain dynamic the MEH-PPV sample has been irradiated with blue light (λ = 480 nm Intensity =3.2x10^{17} photons s^{-1} cm^{-2} $t = 800$ s) in order to achieve a moderate degradation stage. This degraded samples present a typical charge domain size of about 30nm, slightly smaller than the corresponding non-degraded sample and a standard deviation of $\sigma_S = 100$ mV. Fig.2 shows four consecutives frames extracted from a movie (183 seconds/frame) together with the cross-correlation between the first frame and successive frames. Comparing this degraded sample with the non-degraded one, it is found that although in the degraded sample a charge domain dynamic is still present, the corresponding changes occur less frequently and the decay time of the cross-correlation increases from about 3 min to about 15 min. Those results fully support the idea that we are observing hopping dynamics, and that lower conductance is correlated with slower domain dynamics.

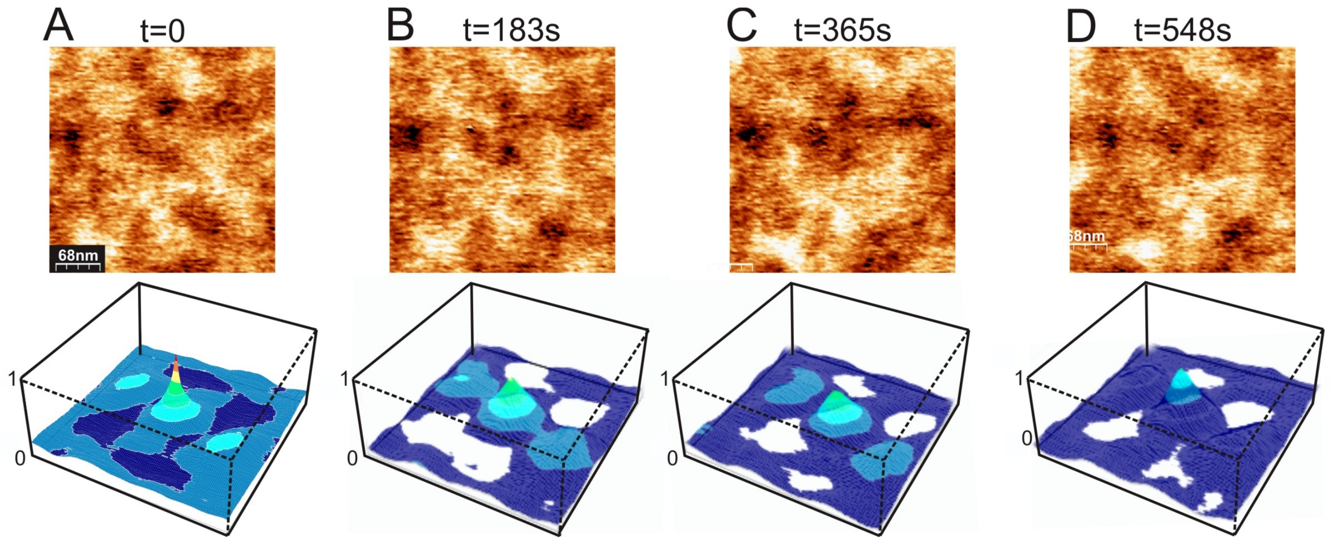

Figure 2. Degraded MEH-PPV sample. Top panel.(A-D) four SP frames from a movie (183 seconds/frame) taken at the times indicated (z scale $\pm$150 mV . Bottom panel. 3D plot of the normalized correlation of the corresponding SP frame with the first frame.

Materials and Methods

Data processing

The well-defined averages of topography and error signal (See Fig. S2 Additional information) prove that the drift correction algorithm aligns the frames of a movie to within a few image points; an accuracy of 2-3 pixels is estimated, which is of the order than the spatial resolution of our experiments. We note that exactly the same alignment protocol is applied to all SFM channels.

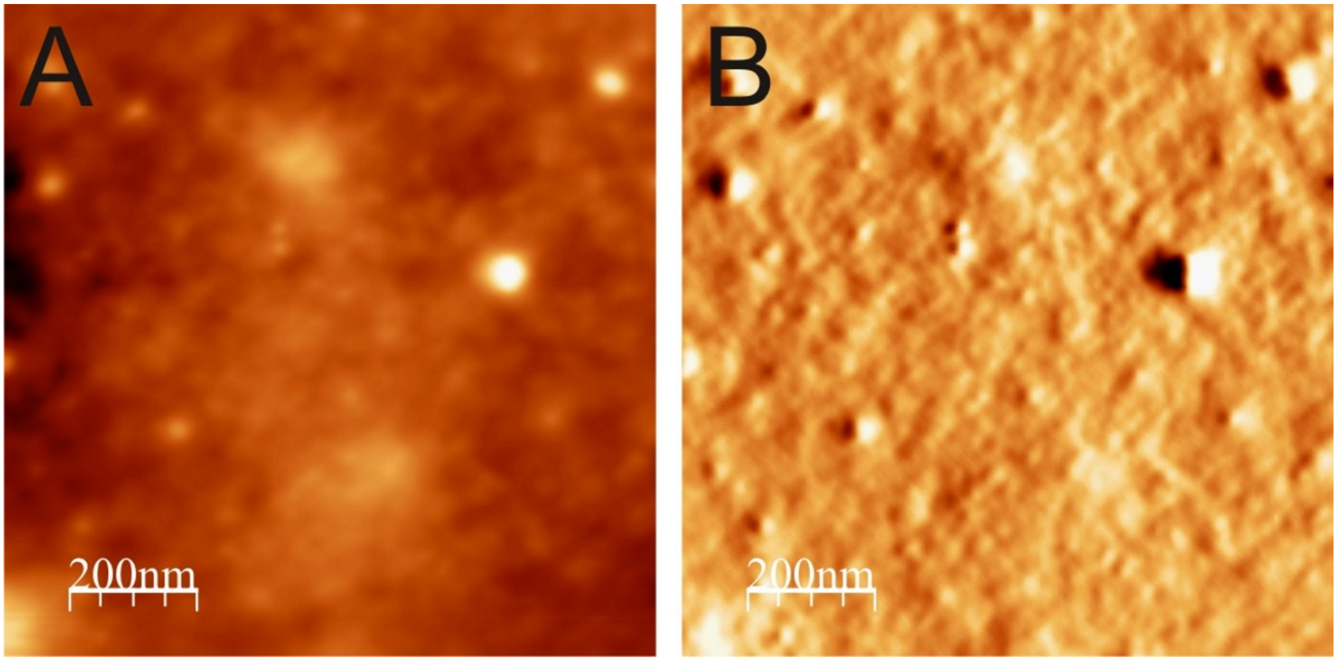

Figure 3. (A) Average topography and (B) average frequency (error signal) image obtained from a movie of 91 frames (20 min/frame).

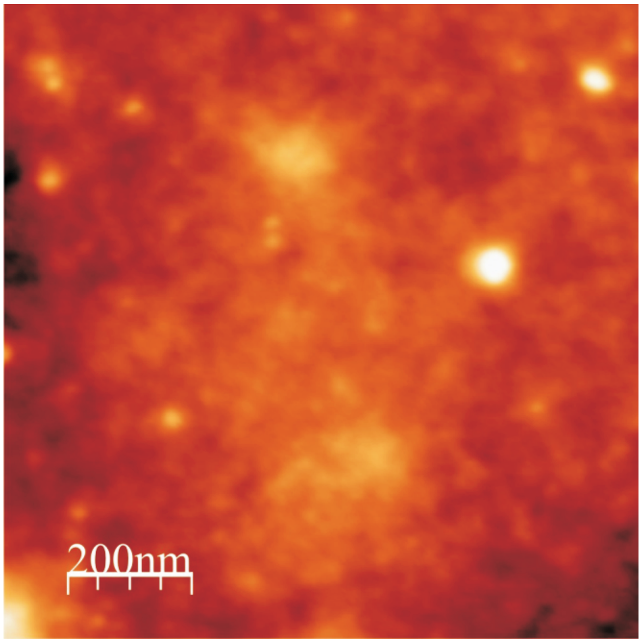

VideoS 1. Topography movie of 91 frames acquired at 20 min/frame (total video time= 1820 min).

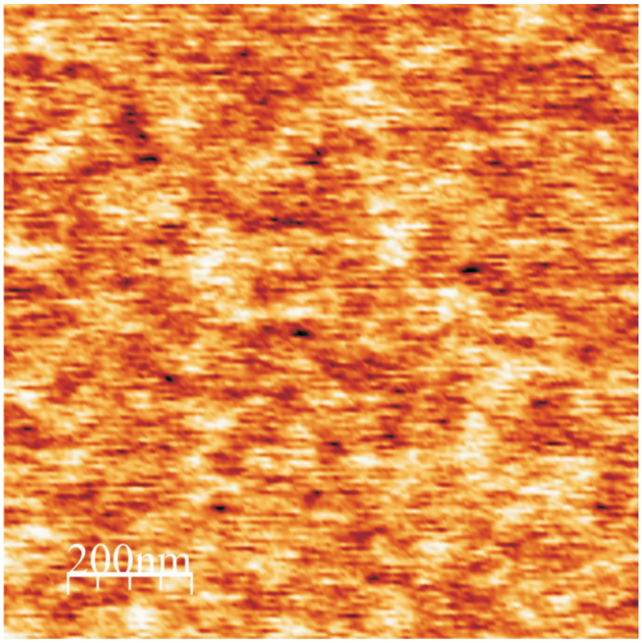

VideoS 2. Surface potential movie of 91 frames acquired at 20 min/frame (total video time= 1820 min).

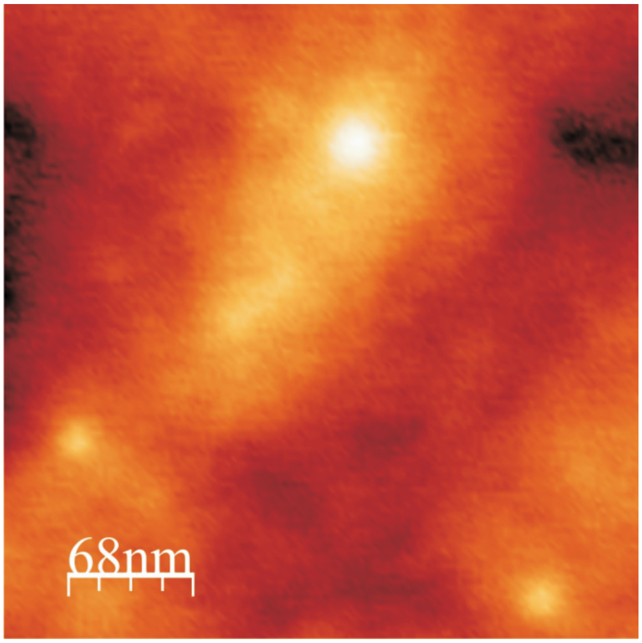

VideoS 3. Topography movie of 40 frames acquired at 1 min/frame (total video time= 40 min).

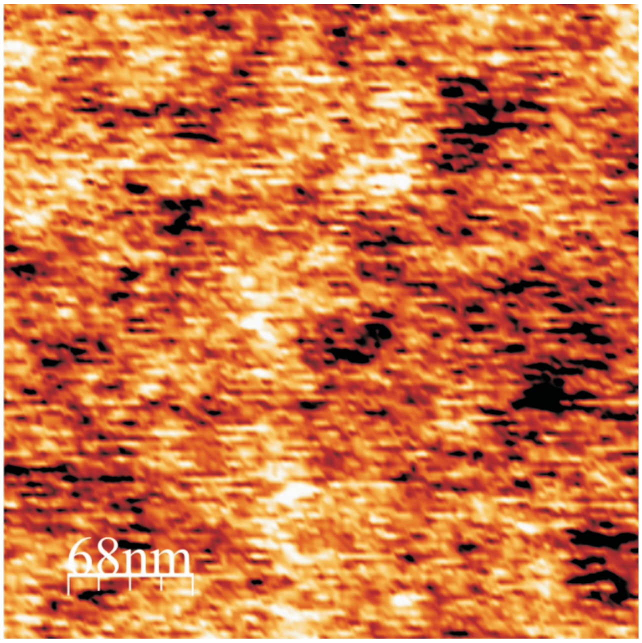

VideoS 4. Surface potential movie of 40 frames acquired at 1 min/frame (total video time= 40 min).

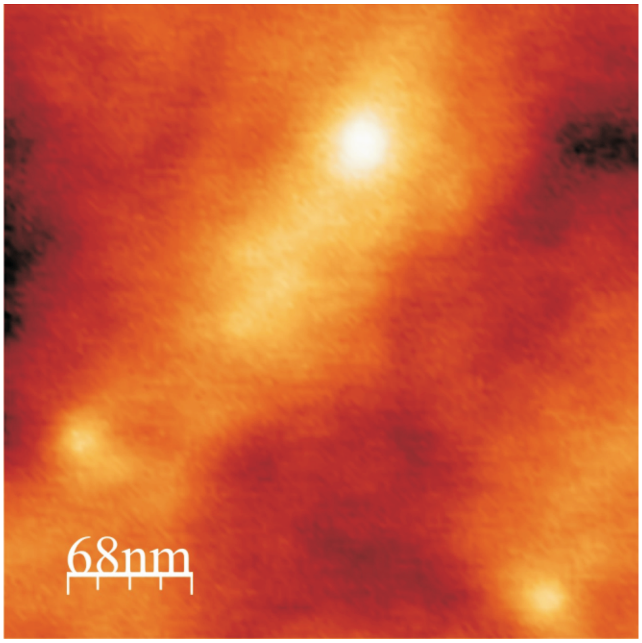

VideoS 5. Topography movie of 80 frames acquired at 2 min/frame (total video time= 40 min).

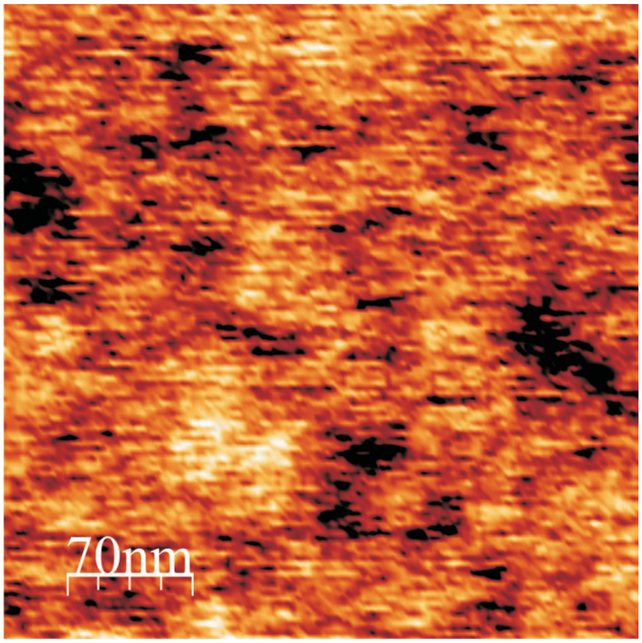

VideoS 6. Surface potential movie of 40 frames acquired at 2 min/frame (total video time= 40 min).
